# Real-World Analysis of the Impact of Radiotherapy on Immunotherapy Efficacy in Non-Small Cell Lung Cancer

**DOI:** 10.3390/cancers13112800

**Published:** 2021-06-04

**Authors:** Amir Onn, Teodor Gottfried, Amos Stemmer, Sarit Appel, Yaacov R. Lawrence, Damien Urban, Tamar Beller, Sameh Daher, Jair Bar

**Affiliations:** 1 Institute of Pulmonology, Sheba Medical Center, Ramat Gan 5262000, Israel; Amir.Onn@sheba.health.gov.il; 2 Sackler School of Medicine, Tel Aviv University, Tel Aviv 6997801, Israel; amos@mail.tau.ac.il (A.S.); Sarit.Appel@sheba.health.gov.il (S.A.); Yaacov.Lawrence@sheba.health.gov.il (Y.R.L.); Damien.Urban@sheba.health.gov.il (D.U.); 3 Institute of Oncology, Sheba Medical Center, Ramat Gan 5262000, Israel; Teodor.Kuznetsov@sheba.health.gov.il (T.G.); Tamar.Beller@sheba.health.gov.il (T.B.); sameh.daher@sheba.health.gov.il (S.D.); 4 Radiation Oncology Department, Sheba Medical Center, Ramat Gan 5262000, Israel

**Keywords:** real-world data, radiation treatment, immune sensitization, radiation doses, radiotherapy fractionation, radiation targets

## Abstract

**Simple Summary:**

Immunotherapy (IO) and radiotherapy (XRT) are two of the most important treatment modalities in metastatic non-small cell lung cancer. There is data to suggest that XRT can enhance the IO’s efficacy. However, little is known regarding how to best combine them. In this retrospective, single-center study, we analyze data of 453 patients who have received various combinations of XRT and IO, or IO alone, to assess the treatment parameters that correlate with longer overall survival (OS). XRT doses between 30 and 40 Gy correlated with longer overall survival, while XRT doses below 10 Gy, fractions of 4.1 to 8 Gy and XRT to the bone correlated with worse overall survival. These results require validation with prospective studies.

**Abstract:**

Background: Immunotherapy (IO) provides a significant benefit for a subgroup of non-small cell lung cancer (NSCLC) patients. Radiotherapy (XRT) might enhance the efficacy of IO. We evaluated the impact of the specifics of XRT treatments on the OS of IO-treated NSCLC patients. Methods: Metastatic NSCLC patients treated with IO were retrospectively identified. Parameters included demographics, tumor characteristics, IO and XRT details. Correlation between the parameters and OS was tested with Cox regression. Results: 453 patients were included. No XRT was given to 167 (36.9%) patients, whereas XRT prior and after IO had 182 (40.2%) and 104 (22.9%) patients, respectively. XRT total doses between 30 and 40 Gy had better overall survival (OS) compared to non-irradiated patients (hazard ratio (HR) 0.5, 95% CI 0.25–1.0, *p* = 0.049). Worse outcome was seen with total doses ≤ 10 Gy (HR 1.67, 95% 1.13–2.5, *p* = 0.01), XRT fractions of 4.1–8 Gy (HR 1.48, 95% CI 1.05–2.1, *p* = 0.027) and XRT to the bone (HR 1.36, 95% CI 1.01–1.8, *p* = 0.04). Several clinical parameters correlated with OS in the univariate analysis of the IO-treated patients. While, in the multivariate analysis, only ECOG-PS, treatment line, type of IO, albumin and NLR remained statistically significant. Conclusion: Specific doses, fractions and sites of XRT correlated with the OS of IO-treated NSCLC patients in the univariate analysis, although not in the multivariate analysis.

## 1. Introduction

Immunotherapy (IO) has revolutionized oncology, with a marked impact on the treatment of lung cancer. Checkpoint inhibitors, specifically antibodies targeting the programed cell-death-1 (PD-1) and its ligand (PD-L1) interactions, have become part of the standard of care for non-small cell lung cancer (NSCLC). Pembrolizumab is now indicated for most patients as first-line treatment for metastatic disease, either as a single-agent or in combination with chemotherapy, depending on the expression levels of tumor PD-L1 protein [1,2]. Atezolizumab is also approved as first-line therapy alone or in combination with chemotherapy [3,4]. Cytotoxic T lymphocyte protein 4 (CTLA-4) is an additional target of immunotherapy, currently tested also in NSCLC in combination with anti-PD-1 antibody [5]. Anti-PD-1 and anti-PD-L1 antibodies have also been utilized for several years now as second-line treatments for advanced NSCLC. Although a higher rate of response is seen with IO compared to chemotherapy regimens and the duration of the response is longer, the majority of patients still succumb to their disease. Ongoing studies aim to improve on these results by various immune-modulation approaches and novel drugs. The number of possible treatment tactics and combinations is immense, as reflected by the huge expansion in the number of ongoing clinical trials in this field [6]. 

Ionizing radiation (XRT), another pillar of the anti-cancer therapeutic options, can impact the immune system and is suggested to synergize with IO. The PACIFIC study demonstrated the benefit from addition of IO following chemo-radiotherapy treatment on stage III non-resectable NSCLC [7]; however, this study does not directly prove synergism between the different modalities. Anecdotal reports of abscopal responses to radiotherapy suggest an induction of a systemic anti-tumor immune response by localized radiotherapy [8]. Mice models demonstrate the abscopal effect to be dependent on the immune system [9], possibly related to the activity of the TP53 tumor suppressor [10] and the TGF-beta signaling pathway [11]. Radiation might convey the signals necessary to induce immune cells through exosomes carrying broken DNA [12], in turn activating stimulator of interferon genes (*STING*) and interferon signaling. Additional studies suggest radiation to induce enhanced antigen presentation, immune cell infiltration and activation [13]. Retrospective analyses of NSCLC patients’ data may support the role of XRT as synergistic with IO. Some studies indicate XRT enhances the efficacy of IO if given prior to IO [14,15], while other reports support concurrent XRT and IO [16], and some do not interrogate at all the impact of the specifics of XRT treatments [17]. As retrospective studies, various types of possible bias reduce the reliability of this data. Very few clinical studies have been reported so far to support the potential therapeutic role of XRT beyond the radiation field, mostly single-arm studies of IO-treated patients [18,19]. A recent phase 2 randomized study comparing pembrolizumab with or without prior stereotactic body radiation (SBRT) did not reach its primary goal but did demonstrate an increased response rate in the experimental arm, specifically among the PD-L1-negative tumors [20]. A large number of ongoing studies are investigating XRT addition to IO [21] (focusing on a potential systemic impact of XRT, we do not relate here to studies targeting all sites of oligo-metastatic disease); no clear results have been reported thus far. Importantly, the complexity of the molecular and cellular mechanisms involved point to the potential of an antagonistic effect of XRT on IO in some circumstances [13,22]. An important consideration is the multiple manners in which XRT can be integrated with IO treatment, regarding timing, doses, fractionation and sites to irradiate; each of these parameters is likely to impact the immune system differently. Aiming to gain further insight into the ways to potentially harness XRT as an immune-sensitizing treatment, we turned to available real-world data. We provide here a detailed analysis of a large set of advanced IO-treated NSCLC patients, aiming to further evaluate the potential role of XRT and specific radiotherapy parameters as synergistic with IO. 

## 2. Materials and Methods

### 2.1. Patients 

Metastatic NSCLC patients were included if they have received at least one IO treatment for advanced disease between January 2015 and September 2019. Patients were retrospectively identified from the working database of the lung unit at the Institute of Oncology at Sheba Medical Center. Data extracted included demographics (sex, age), details about the cancer (histology, mutational analysis done as part of the standard-of-care), the type of immunotherapy administered (single agent, combined with chemotherapy or with another type of immunotherapy) and the specific immunotherapy drug given and the line of immunotherapy treatment. Radiotherapy treatment details were extracted from the administrative records and clinical charts of the Department of Radiotherapy at Sheba Medical Center. For patients that have received more than one XRT course, the course that occurred closest to the initiation of IO was chosen for further analysis. Additional parameters collected concerning the patients’ general condition at the time of initiating IO were the Eastern Cooperative Oncology Group performance status (ECOG-PS), albumin levels and neutrophil-to-lymphocyte ratio (NLR). Blood test data were included if conducted within a month prior to initiation of immunotherapy. 

### 2.2. Statistics

Categorical and ordinal variables were tested for significance by Chi-square test. Categoric parameters included sex, histology, presence of mutations (none, Kirsten rat sarcoma viral oncogene homolog *(KRAS),* Epidermal growth factor receptor *(EGFR)* and others), type of immunotherapy, specific immunotherapy drug used and blood albumin (below or above the lower limit of the norm). Timing of XRT initiation relative to immunotherapy was interrogated as categorical, comparing any timing prior to IO initiation to any timing after that point. Additional analyses included radiotherapy-treated patients at various pre-specified time windows (within a period of one, three or six months before or after initiation of IO). Target organs of XRT were grouped as lung and mediastinum, bone, cranium (not including stereotactic radiosurgery, SRS), gastro-intestinal and soft tissue. SRS-treated patients were excluded from the analysis due to small numbers.

Ordinal variables included ECOG-PS, total radiotherapy dose and radiotherapy fraction size. Categories of total radiotherapy dose were arbitrarily defined in 10 Gray (Gy) steps, the highest dose group being above 50 Gy. The mode of radiotherapy (SBRT or regular external beam radiotherapy) was not evaluated separately in this study. Fraction sizes were examined in arbitrary cohorts of up to 2 Gy, 2.1–4 Gy, 4.1–8 Gy and larger than 8 Gy. As a sensitivity analysis, the total XRT dose and fraction size were also investigated as continuous variables. 

All continuous parameters were tested for variance homogeneity by Levene’s test. Comparisons were done by Student’s t-test, or ANOVA for more than two groups. In the event the distribution is found to be significantly different than normal, Welch’s t-test was used. Continuous variables included age, NLR and treatment line of IO. 

The primary endpoint of this study was overall survival, calculated from the initiation of IO till death or censured at last follow-up. All of the above parameters were tested for correlation with overall survival (OS) by Cox regression as a univariate analysis, followed by multivariate analyses. Multivariate analysis included age, sex, all XRT parameters and any additional factor that demonstrated significance in the univariate analysis. Significance was defined as a *p*-value of 0.05 or lower. 

Blood test results (albumin and NLR) were not available for the entire study cohort. Therefore, multivariate analyses were done both without these parameters, including the entire cohort, as well as with these parameters, thus including a smaller number of patients. 

### 2.3. Ethics

The study was approved by the institutional ethics committee (approval #8993-11-SMC).

## 3. Results

### 3.1. Patients Characteristics

A total of 563 lung cancer patients received IO during the study period. Of these, 85 patients were excluded for any of the following reasons: a diagnosis different from NSCLC; received IO but not as treatment for metastatic disease (e.g., durvalumab for stage III NSCLC); or treated under a blinded clinical trial. In addition, 25 patients that received SRS were excluded, leaving 453 patients that were included in the study. Blood tests of the albumin levels and NLR were available for 374 patients.

No XRT was delivered for 167 (36.9%) of the study cohort. XRT was given prior to IO for 182 (40.2%) of the cohort and 104 (22.9%) received XRT after IO initiation. XRT was initiated before and completed after IO initiation for 21 patients out of the 182 patients in the XRT-prior-to-IO group. 

As can be seen (Table 1), the three XRT-timing groups differed in some of the examined parameters. Specifically, ECOG-PS was better, albumin was higher and NLR was lower for the group receiving XRT after IO. NLR was also lower for the non-irradiated group. Low total XRT doses were more common after IO compared to the XRT-before-IO group. A small fraction size was more common before IO. 

At a median follow-up of 23 months (IQR, 9–35), 280 (74.9%) of the patients died. The median OS was 10 months (95% CI 8–12). The characteristics of the patients included in this study, according to the timing of XRT relative to IO initiation, are presented in Table 1. 

### 3.2. XRT Timing Impact on IO-Treated Patients

**Figure 1:**
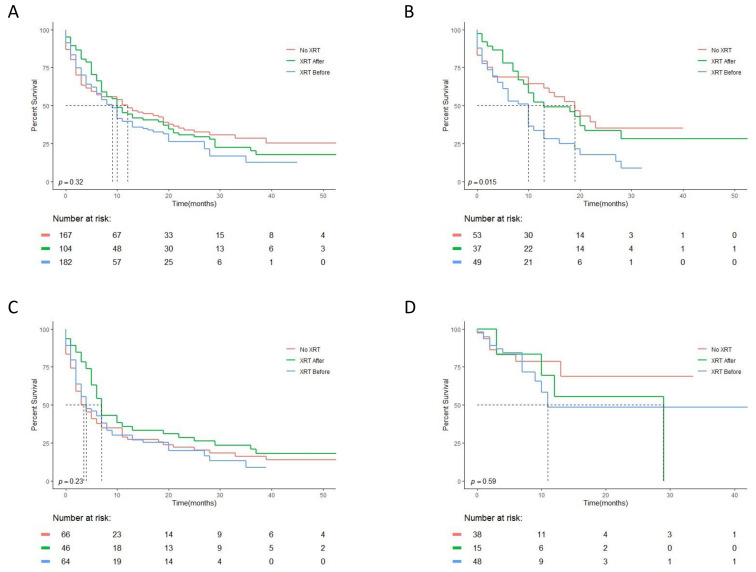
Impact of XRT and its timing relative to IO initiation. Overall survival of the different timing groups (no XRT, XRT after IO, XRT before IO) in (**A**) all of the study patients, (**B**) patients treated with pembrolizumab as a single agent, (**C**) patients treated with nivolumab as a single agent and (**D**) patients given chemotherapy and IO combination therapy.

Following a previous report of a beneficial impact of XRT given prior to pembrolizumab [15], we evaluated the survival of patients in our cohort, comparing those that did not receive any XRT, those receiving XRT prior to IO and those receiving XRT after IO initiation. As can be seen in Figure 1A, no difference was seen between these groups. To further evaluate our data, we compared these three timing groups among patients that received specifically pembrolizumab alone. In contrast to a previous report [15], the pembrolizumab-treated patients who received XRT prior to pembrolizumab demonstrated significantly shorter survival compared to the non-irradiated patients, as well as compared to those receiving XRT after pembrolizumab initiation (Figure 1B). All other IO subgroups tested (nivolumab treated, Figure 1C, and IO and chemotherapy combination—Figure 1D) did not demonstrate a statistically significant difference in survival depending on XRT administration and its timing. However, survival curves of patients given XRT prior to IO were mostly below the other survival curves in all cases. Atezolizumab-treated and IO–IO-treated patients constituted groups not large enough to allow valid comparisons of the XRT timing sub-groups. 

**Figure 2:**
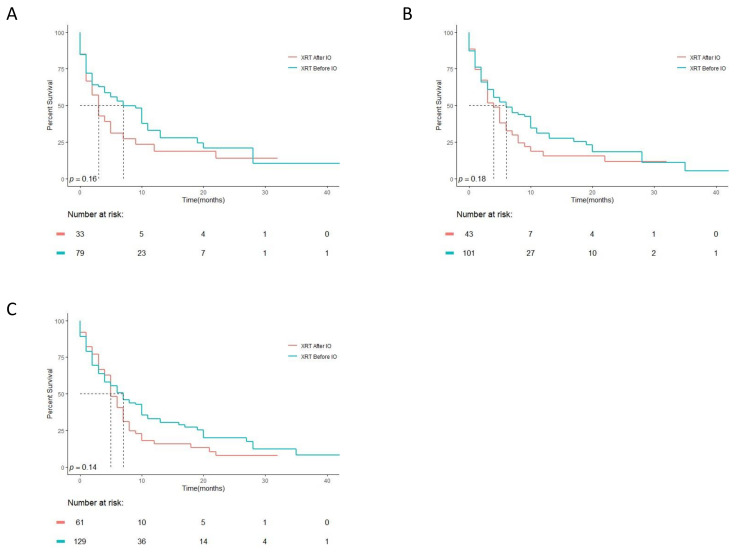
Kaplan–Meier overall survival comparison of patients given XRT before or after IO initiation, within one month before or after IO initiation (**A**), within 3 months (**B**) or within six months before or after IO initiation (**C**).

We speculated that if XRT impacts the efficacy of IO, it’s effect would be most evident when given around the time of IO initiation. We therefore compared patients that initiated XRT treatment prior to IO initiation to patients that received XRT after initiation of IO, but only within a limited time frame of one month before or after IO initiation. As seen in Figure 2A, in this comparison, a non-significant trend for better outcome can be seen for patients that received XRT prior to IO initiation. Similar results were seen when larger time windows were examined up to six months before or after IO initiation (Figure 2B,C).

### 3.3. XRT Parameters Impact on Outcome of IO-Treated Patients

**Figure 3:**
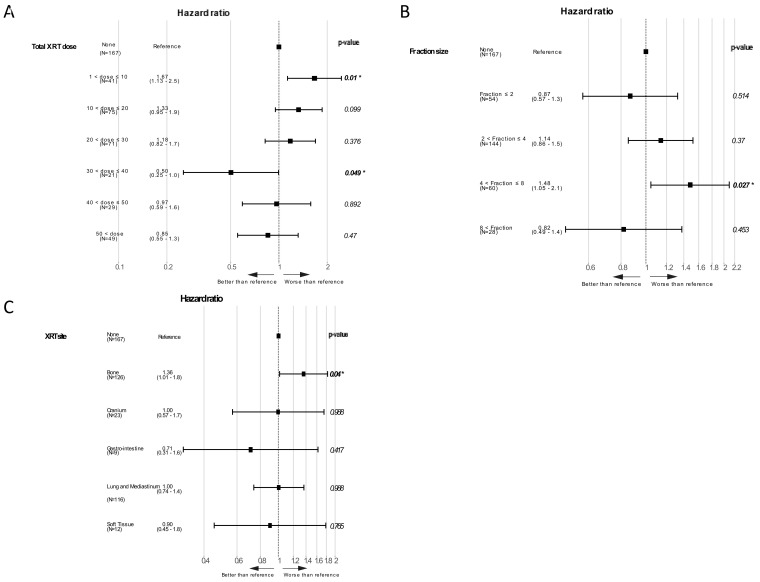
Forest plot demonstrating the overall survival hazard ratio and 95% CI of patients, (**A**) grouped by the total dose received, (**B**) grouped by fraction size and (**C**) grouped by the site irradiated. In all cases, each sub-group is compared to the non-irradiated group, the hazard ratio with 95% CI are depicted and the *p*-value presented. *p*-values smaller than 0.05 are in bold.

We reasoned that the inconsistency of our results regarding XRT timing relative to IO initiation might stem from the variability of the XRT regimens utilized as well as the heterogeneity of the study population. We therefore evaluated additional XRT parameters of the treatment given. We examined the impact of total dose of XRT given on patients’ survival. For this goal, patients were grouped by the total XRT dose in steps of 10 Gy and each group was compared to non-irradiated patients. As seen in Figure 3A, patients treated with doses between 30 and 40 Gy had a better outcome than non-irradiated patients (HR 0.5, 95% CI 0.25–1.0), with borderline significance (*p* = 0.049). In contrast, patients treated with doses of 10 Gy or less had a worse outcome than non-irradiated patients (HR 1.67, 95% 1.13–2.5, *p* = 0.01). We next examined the prognostic value of fraction size, with patients grouped by this parameter compared to the non-irradiated group. As seen in Figure 3B, patients receiving fractions in the range of 4.1 to 8 Gy had a worse outcome than non-irradiated patients (HR 1.48, 95% CI 1.05–2.1, *p* = 0.027). No dose-per-fraction-dependent size effect was seen in this analysis, as the group of patients receiving fractions larger than 8 Gy did not have a worse outcome than the non-irradiated group; notably, the size of this group was relatively small. Total XRT dose was not correlated with survival when tested as a continuous variable (HR 0.99, 95% CI 0.99–1.00, *p* = 0.150), similar to fraction size as a continuous variable (HR 1.01, 95% CI 0.98–1.04, *p* = 0.436). The distribution of total doses and fraction sizes as administered in different number of fractions is demonstrated in Supplementary Table S1. We next evaluated the role of the site irradiated, comparing patients irradiated at various sites with the non-irradiated group. Patients receiving bone XRT had a significantly worse outcome than non-irradiated patients (HR 1.36, 95% CI 1.01–1.8, *p* = 0.04; Figure 3C). 

### 3.4. Patient and Treatment Characteristics’ Impact on Outcome of IO-Treated Patients

We next proceeded to evaluate all of the parameters available for these patients as potential prognostic biomarkers in a univariate analysis. As mentioned above and as seen in Table 2, total XRT dose, size of fractions (both as ordinal variables, not as continuous variables) and irradiated site correlated with OS of the IO-treated patients. In addition, ECOG-PS, treatment-line of IO (i.e., whether given as 1st line or at later lines of treatment), immunotherapy type given, albumin levels and NLR were all significantly and highly correlated with OS of IO-treated patients. As a sensitivity analysis, albumin was examined also as a continuous variable, found to be also significantly correlated with survival (HR 0.31, 95% CI 0.25–0.39, *p* < 0.001). Timing of the XRT, as well as age, sex, histology or mutations of the tumors were not significantly correlated with survival. 

The impact of different tumor histologies was further examined by Kaplan–Meier analysis, demonstrating largely overlapping survival curves when the entire cohort was included, but some separation and a trend for better outcome for squamous cell cancer patients was seen when only irradiated patients were included (Supplementary Figure S1). A similar analysis for type of IO given can be seen in Supplementary Figure S2, demonstrating a trend of a better outcome for the IO–chemotherapy combination and for the atezolizumab-treated patients, both for the entire cohort as well as for the irradiated-only patients. Impact of the site of radiotherapy is presented in Supplementary Figure S3. 

**Figure 4:**
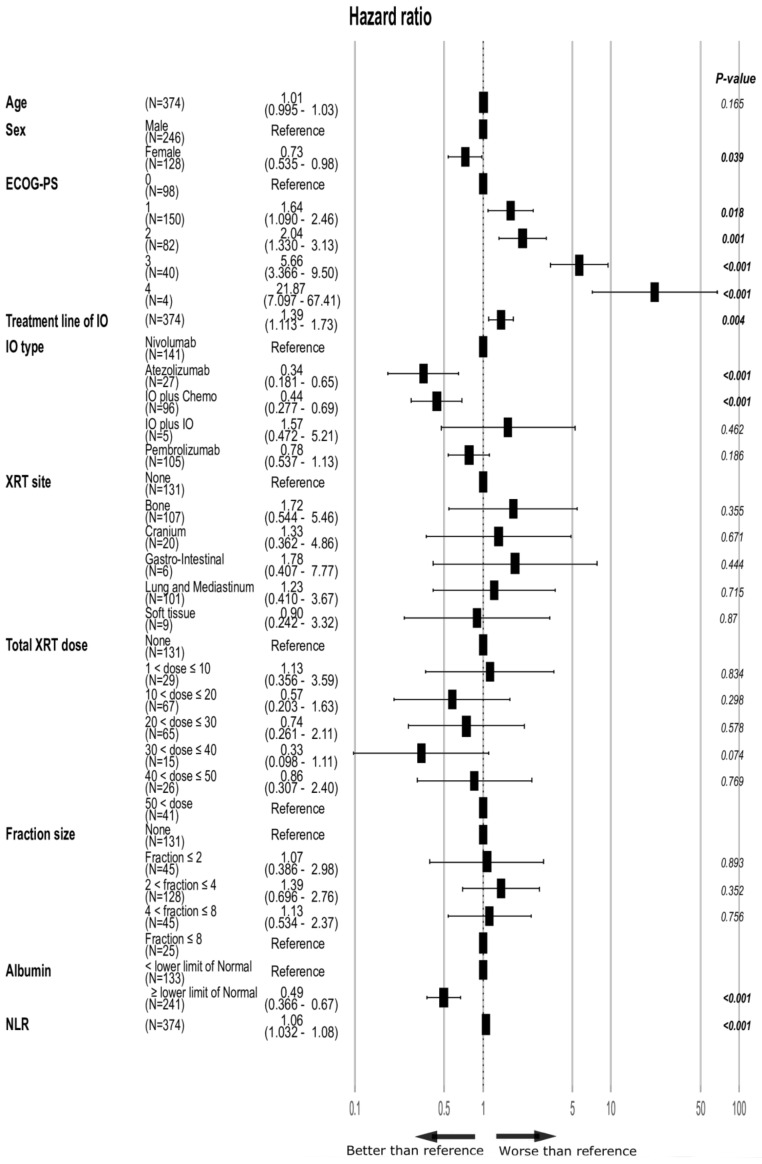
Forest plot of the multivariate evaluation of the factors affecting OS (*n* = 374, excluding patients with no blood test results available). Total XRT dose and fraction size are presented in Grays. *p*-values of statistically significant hazard ratios are in bold. XRT timing—related to timing relative to IO initiation; ECOG-PS: performance status; XRT: radiotherapy; IO: immunotherapy; Chemo: chemotherapy; Tx: treatment; NLR: neutrophil-to-lymphocyte ratio.

The role of XRT site, total XRT dose, XRT fraction size and timing of XRT, as well as ECOG-PS, albumin, NLR, treatment line and type of IO were interrogated by multivariate analyses (Figure 4). Here, only ECOG-PS, treatment line of IO, type of IO, albumin and NLR remained significantly correlated with OS. Importantly, none of the XRT-related parameters were associated with OS in this analysis. In a sensitivity analysis including all patients, disregarding the blood test results (Supplementary Figure S4), similar results were found, except for a significant benefit for the female sex (HR 0.72, 95% CI 0.55–0.95, *p* = 0.019) seen here, but which was not significant when the blood test results were included in the analysis. 

## 4. Discussion

This report is one of the largest and most detailed retrospective analyses of IO-treated, advanced NSCLC patients. We focused on the impact of XRT on these patients and initially aimed to reproduce previously reported results indicating a benefit for XRT administration prior to IO. The earlier study we have attempted to validate was a retrospective analysis of data from a prospective study, but included only 98 patients, without any correction for confounding factors [15]. Besides details of the XRT treatments, we included in the present analysis clinical, pathologic and laboratory data that are potentially prognostic for survival of advanced NSCLC. Importantly, when all the available parameters were taken into account, no XRT characteristic remains significantly associated with survival; therefore, we failed to validate previously published results [15]. In contrast, general factors, such as ECOG-PS [23,24], albumin [25] and NLR [26,27,28], which were previously reported to be significantly associated with survival, remain so, even in this highly heterogeneous cohort of patients, attesting to the validity of our results. The potential benefit of XRT should be investigated further by well-designed, randomized interventional prospective studies. 

Numerous studies are currently assessing XRT in combination to IO treatments in various scenarios. The majority of these studies include a similar XRT regimen for all study participants and are testing the role of addition of IO to this treatment [29]. Only a few large randomized studies include XRT as the investigated intervention. Several studies are testing the impact of an XRT plan where the aim is eradiation of all sites of disease [30,31] (e.g., NCT03867175); we believe such studies are not directly testing XRT as an immune modulator. Phase III studies that test XRT to one or more sites of poly-metastatic disease include NIRVANA-LUNG (NCT03774732) and LONESTAR (NCT03391869). The COSINR study compares SBRT given prior to IO or concurrent with IO for metastatic lung cancer (NCT03223155). The ARCHON-1 study is testing durvalumab with accelerated vs. conventional fractionation of XRT with durvalumab for non-metastatic unresectable lung cancer (24 patients planned; NCT03801902). The paucity of studies assessing the details of the XRT treatments that may prove synergistic with IO prompted us to perform the analysis described in this manuscript. Our findings of a correlation of specific doses, fractionations and irradiated sites with survival in univariate analysis can potentially direct future studies in the field. We suspect that without evaluation of the characteristics of the XRT regimen that optimally induce the immune system, the efforts invested into large-scale clinical trials may be futile. 

Importantly, some of our results contradict commonly accepted but unproven paradigms, such as the use of eight Gy XRT fractions in combinations with IO [32]. This paradigm was proven in mice; for example, a study demonstrating that three fractions of eight Gy had a better response than five fractions of six Gy in inducing anti-tumor immunity in combination with anti-CTLA-4 antibodies [33]. However, the physical and immunological impacts of XRT for mice and humans are not the same [34], making it difficult to relay on pre-clinical evidence for choosing the optimal manner to treat people. Some data from human studies besides our own also contradict the mice data. For example, a regimen of 35 Gy in 10 fractions administered concurrently with GM-CSF achieved a 27% abscopal response rate in a set of 41 patients with advanced solid tumors [18]. A set of 47 melanoma patients treated with ipilimumab and radiotherapy were analyzed retrospectively, searching for XRT parameters that correlate with abscopal response—the only parameter found to be relevant was a dose of less than 3 Gy per fraction [35]. Another retrospective analysis of 69 NSCLC patients treated by chemotherapy, of which 45 patients got palliative rads (30 Gy in 10 fractions or 20 Gy in 5 fractions), demonstrated better survival for the irradiated patients [17]. The pro-immunogenic impact of lower doses of XRT is supported by a number of pre-clinical and clinical observations [36]. Other findings we report, such as a negative outcome associated with XRT to an osseous lesion, may mirror impressions of other investigators [32]. A better outcome of a IO–chemotherapy combination as what we found in our multivariate analysis has been recently shown using real-world data, although correction for confounding factors was not included in that report [37]. The better outcome we found for the atezolizumab-treated patients compared to other immune checkpoint inhibitors is unexpected, but considering the small number of such patients in this cohort, this result should be interpreted cautiously. 

Importantly, the cohort we have described, consisting of real-world advanced NSCLC patients, demonstrates, as expected, a worse outcome than reported in clinical studies, with a median OS of 10 months. Specifically, more than 30% of our cohort had an ECOG-PS higher than one; such patients would not have been included in any clinical trial. The parameters of XRT that correlated with worse outcome are also those of palliative XRT treatments administered commonly to poor PS patients. For example, patients in a general poor condition are commonly treated by a single large fraction of eight Gy as palliative treatment. Indeed, a total dose of less than 10 Gy and a fraction size of 4–8 Gy were correlated with poor outcome in our study. Accordingly, when corrected for ECOG-PS and other prognostic factors, the impact of this radiation dose and fraction size was not significant. The complex interplay between the patient’s general condition, the burden of disease, the activity level of the patient’s immune system and the impact of the IO and XRT treatments is far from being elucidated at this time.

The main limitation of this study is its retrospective nature. Similar to all real-world studies, it was hampered by missing data and the obvious bias of treatment assignment being dictated by clinical need. Another potential caveat of our study is the possibility of missing data due to patients receiving XRT treatment in others centers, although this is unlikely considering our center is a tertiary oncology referral center. Moreover, the pertinent information regarding the clinical, pathologic and treatment parameters were successfully collected from the entire identified cohort, besides the blood test results, which were available only for 82.6% of the cohort (374 of 453 patients). The limitation of the study being a single-center study actually facilitated the complete capture of data from the local medical records. Another factor to consider in the analysis of our results is a potential for lead-time bias when comparing patients that got XRT before versus after IO initiation. However, since no significant difference was seen in favor of XRT before IO, the impact of this bias is minor in our study, but might be playing a role in other retrospective analyses [15].

## 5. Conclusions

This study points at details of XRT regimens that might synergize with IO, such as a total dose of 30–40 Gy. Importantly, our study identifies XRT characteristics that are possibly antagonistic with IO, such as a total dose of 10 Gy or less, a fraction size of 4.1 to 8 Gy and irradiation to bone lesions. After correction for potential confounding factors using multivariate regression, none of the tested parameters of XRT remains statistically significantly when correlated with survival. However, our results should be taken into consideration in the design of future studies and in the analysis of the ongoing studies investigating the role of XRT in patients treated with IO. 

## Figures and Tables

**Table 1:** Characteristics of the patients included in the study.

Parameters	All Patients	No XRT	XRT before IO	XRT after IO	*p*-Value
N (%)	453 (100)	167 (100)	182 (100)	104 (100)	
Age (years) median (range)	67 (34–96)	69 (38–96)	67 (43–89)	66 (34–83)	0.0369 *
Sex-male (%)	296 (65.3)	107 (64.1)	116 (63.7)	73 (70.2)	0.495 ^§^
ECOG-PS—N (%)					**0.025** ^§^
0–1	312 (68.7)	112 (67.1)	117 (64.3)	83 (79.8)	
2–3	137 (30.2)	52 (31.1)	64 (35.2)	21 (20.2)	
4	4 (0.9)	3 (1.8)	1 (0.5)	0	
XRT site—N (%)					**0.049** ^§^
None	167 (36.9)	167 (100)			
Bone	126 (27.8)		86 (47.3)	40 (38.5)	
Cranium	23 (5.1)		10 (5.5)	13 (12.5)	
Gastro-intestinal	9 (2.0)		4 (2.2)	5 (4.8)	
Lung and Mediastinum	116 (25.6)		77 (42.3)	39 (37.5)	
Soft tissue	12 (2.6)		5 (2.7)	7 (6.7)	
Total XRT dose (Gy)—N (%)					<**0.001** ^§^
None	167 (36.9)	167 (100)			
1 < dose ≤ 10	41 (9.1)		19 (10.4)	22 (21.2)	
10 < dose ≤ 20	75 (16.6)		47 (25.8)	28 (26.9)	
20 < dose ≤ 30	71 (15.7)		45 (24.7)	26 (25.0)	
30 < dose ≤ 40	21 (4.6)		8 (4.4)	13 (12.5)	
40 < dose ≤ 50	29 (6.4)		18 (9.9)	11 (10.6)	
50 < dose	49 (10.8)		45 (24.7)	4 (3.8)	
Fraction size (Gy)—N (%)					<**0.001** ^§^
None	167 (36.9)	167 (100)			
Fraction ≤ 2	54 (11.9)		49 (26.9)	5 (4.8)	
2 < fraction ≤ 4	144 (31.8)		85 (46.7)	59 (56.7)	
4 < fraction ≤ 8	60 (13.2)		32 (17.6)	28 (26.9)	
8 ≤ Fraction	28 (6.2)		16 (8.8)	12 (11.5)	
IO—N (%)					0.136 ^§^
Nivolumab	176 (38.9)	66 (39.5)	64 (35.2)	46 (44.2)	
Atezolizumab	32 (7.1)	8 (4.8)	19 (10.4)	5 (4.8)	
IO plus Chemotherapy	101 (22.3)	38 (22.8)	48 (26.4)	15 (14.4)	
IO plus IO	5 (1.1)	2 (1.2)	2 (1.1)	1 (1.0)	
Pembrolizumab	139 (30.7)	53 (31.7)	49 (26.9)	37 (35.6)	
Treatment line of IO—N (%)					0.755 *^,^^‡^
1	226 (49.9)	86 (51.5)	89 (48.9)	51 (49.0)	
≥2	227 (50.1)	81 (48.5)	93 (51.1)	53 (51.0)	
Albumin—gr/dL, mean (95%CI)	3.54 (3.48–3.60)	3.46 (3.35–3.57)	3.51 (3.43–3.59)	3.72 (3.60–3.84)	**0.005** *
NLR—mean (95%CI)	6.77 (6.21–7.34)	6.01 (5.23–6.78)	8.02 (7.06–8.99)	5.52 (4.33–6.71)	<**0.001** *
Histology—N (%)					0.625 ^§^
Adenocarcinoma	309 (68.2)	118 (70.7)	123 (67.6)	68 (65.4)	
Squamous cell	92 (20.3)	31 (18.6)	35 (19.2)	26 (25.0)	
NSCLC-NOS	52 (11.5)	18 (10.8)	24 (13.2)	10 (9.6)	
Mutation—N (%)					0.660 ^§,#^
None	349 (77.0)	125 (74.9)	144 (79.1)	80 (76.9)	
*KRAS*	43 (9.5)	19 (11.4)	14 (7.7)	10 (9.6)	
*EGFR*	31 (6.8)	11 (6.6)	15 (8.2)	5 (4.8)	
*ALK*	2 (0.4)	0	1 (0.5)	1 (1.0)	
*BRAF*	13 (2.9)	6 (3.6)	3 (1.6)	4 (3.8)	
*c-MET*	6 (1.3)	2 (1.2)	3 (1.6)	1 (1.0)	
*ROS1*	5 (1.1)	1 (0.6)	2 (1.1)	2 (1.9)	
Other	4 (0.9)	3 (1.8)	0	1 (1.0)	

*p*-value relates to the comparison between the three following groups: no XRT, XRT before IO, or XRT after IO. Significant *p*-values are in bold. ^§^ Chi-square test. Where irrelevant for the non-irradiated group, Chi-square comparisons were between the groups of XRT before or after IO. * ANOVA test. Rows where one of the values is zero were not included in the Chi-square tests. ^‡^ Presented as categories, but analyzed as a continuous variable. ^#^ Categories analyzed were ‘None, ‘*KRAS’*, ‘*EGFR’* and all the rest. XRT: radiotherapy; IO: immunotherapy; ECOG-PS: performance status at initiation of IO; NLR: neutrophil-to-lymphocyte ratio; NOS: non-otherwise specified; Tx: treatment; Gy: Gray.

**Table 2:** Univariate analysis of the clinical and pathological parameters’ impact on OS of the IO-treated patients.

Parameters	HR (95% CI)	*p*-Value
Age	1.0 (0.99–1.0)	0.196
Sex (men—reference)	0.81 (0.63–1.04)	0.110
ECOG-PS		
0	Reference	
1	1.77 (1.29–2.42)	<**0.001**
2	3.03 (2.15–4.27)	<**0.001**
3	6.37 (4.19–9.68)	<**0.001**
4	31.81 (11.15–90.79)	<**0.001**
XRT site		
None	Reference	
Bone	1.36 (1.01–1.83)	0.040
Cranium	0.99 (0.57–1.75)	0.988
Gastro-intestine	0.71 (0.31–1.62)	0.417
Lung and Mediastinum	1.00 (0.74–1.36)	0.988
Soft Tissue	0.90 (0.45–1.79)	0.765
Total XRT dose (Gray)		
None	Reference	
1< dose ≤ 10	1.66 (1.13–2.46)	**0.010**
10 < dose ≤ 20	1.33 (0.95–1.86)	0.099
20 < dose ≤ 30	1.18 (0.82–1.69)	0.376
30 < dose ≤ 40	0.50 (0.25–0.99)	**0.048**
40 < dose ≤ 50	0.97 (0.59–1.58)	0.891
50 < dose	0.85 (0.55–1.31)	0.470
Fraction size (Gray)		
None	Reference	
Fraction ≤ 2	0.87 (0.57–1.32)	0.513
2 < Fraction ≤ 4	1.14 (0.85–1.52)	0.370
4 < Fraction ≤ 8	1.48 (1.04–2.10)	**0.027**
8 < Fraction	0.82 (0.49–1.37)	0.452
XRT Timing		
No XRT	Reference	
XRT after IO	1.20 (0.91–1.58)	0.188
XRT before IO	1.00 (0.74–1.36)	0.990
Timing Cohorts—time window: *		
XRT after IO	Reference	
One month	0.72 (0.44–1.16)	0.179
Three months	0.76 (0.50–1.14)	0.188
Six months	0.77 (0.54–1.09)	0.139
Treatment-line of IO	1.32 (1.15–1.52)	<**0.001**
Albumin		
< lower limit of Normal	Reference	
≥ lower limit of Normal	0.34 (0.26–0.45)	<**0.001**
NLR	1.06 (1.04–1.07)	<**0.001**
Histology		
Adenocarcinoma	Reference	
Squamous cell	1.01 (0.75–1.36)	0.940
NSCLC NOS	1.20 (0.85–1.72)	0.290
Mutation		
None	Reference	
*KRAS*	9.20 (0.60–1.41)	0.706
*EGFR*	1.15 (0.73–1.82)	0.552
*ALK*	2.34 (0.58–9.44)	0.231
*BRAF*	8.71 (0.43–1.76)	0.701
*c-MET*	7.31 (0.27–1.97)	0.536
*ROS1*	1.06 (0-Inf)	0.990
Others	5.25 (0.13–2.11)	0.364
IO type:		
Nivolumab	Reference	
Pembrolizumab	0.71 (0.54–0.92)	0.011
IO plus Chemotherapy	0.36 (0.24–0.54)	<**0.001**
Atezolizumab	0.79 (0.47–1.34)	0.386
IO plus IO	0.56 (0.178–1.75)	0.315

* Each subgroup of XRT timing prior to IO is compared to XRT after IO within a similar time frame. Significant *p*-values are highlighted in bold. ECOG-PS: performance status; Tx: treatment; XRT: radiotherapy; IO: immunotherapy; NLR: neutrophil-to-lymphocyte ratio.

## Data Availability

Research data are stored in an institutional repository and will be shared upon request to the corresponding author and in accordance to the institutional policies.

## Supplementary Materials

The following are available online at https://www.mdpi.com/article/10.3390/cancers13112800/s1, Figure S1: The impact of the histology of the cancer on overall survival, Figure S2: The impact of type of IO on overall survival, Figure S3: Site irradiated; impact on overall survival, Figure S4: Forest plot of the multivariate evaluation of factors effecting survival, including the whole study cohort, Table S1: Number of patients with the specific combination of fraction number with either total dose or faction size.
