# Supplementary material for: Real-World Analysis of the Impact of Radiotherapy on Immunotherapy Efficacy in Non-Small Cell Lung Cancer
Source: Cancers (Basel). 2021 Jun 4;13(11):2800. doi: 10.3390/cancers13112800 (PMC8200093; doi:10.3390/cancers13112800)

Supplementary Figure S1: The impact of the histology of the cancer on overall survival by Kaplan-Meier analysis, in the entire study cohort (A) or only among irradiated patients (B). Adeno: adenocarcinoma. NOS: non-other specified NSCLC.

A

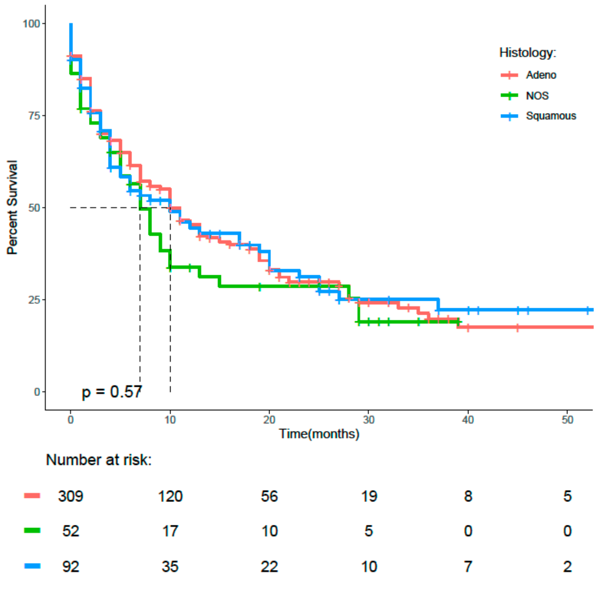

B

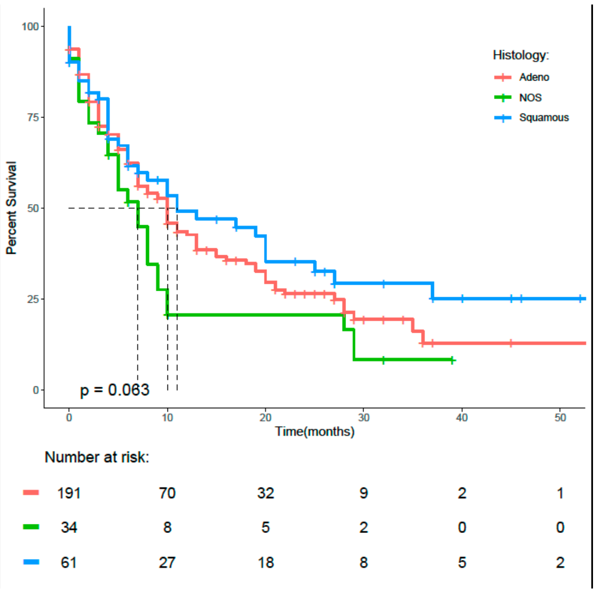

Supplement: Supplementary file 1 [file cancers-13-02800-s001.zip › Supplementary Figure S1.pdf]
