# Supplementary material for: Real-World Analysis of the Impact of Radiotherapy on Immunotherapy Efficacy in Non-Small Cell Lung Cancer
Source: Cancers (Basel). 2021 Jun 4;13(11):2800. doi: 10.3390/cancers13112800 (PMC8200093; doi:10.3390/cancers13112800)
Supplement: Supplementary file 1 [file cancers-13-02800-s001.zip › Supplementary Figure S3.pdf]

Supplementary Figure S3: Site irradiated; impact on overall survival, Kaplan Meier analysis.

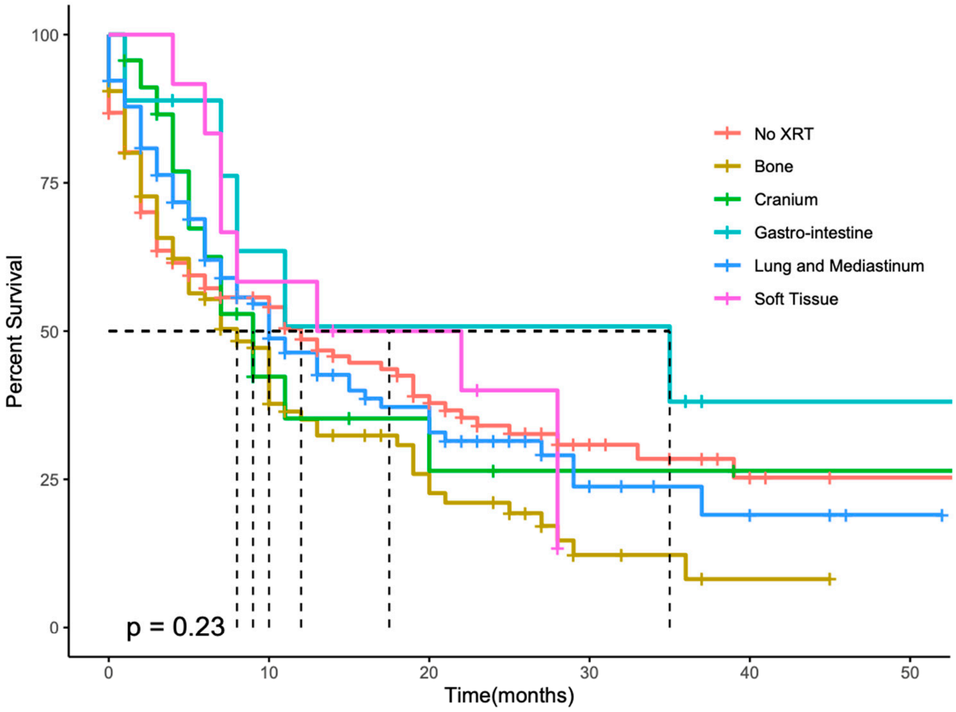

Number at risk:

167	67	33	15	8	4
126	40	16	4	1	0
23	6	4	2	1	1
9	5	4	4	1	1
116	47	26	9	4	1
12	7	5	0	0	0
