# Supplementary material for: Real-World Analysis of the Impact of Radiotherapy on Immunotherapy Efficacy in Non-Small Cell Lung Cancer
Source: Cancers (Basel). 2021 Jun 4;13(11):2800. doi: 10.3390/cancers13112800 (PMC8200093; doi:10.3390/cancers13112800)

Supplementary Figure S4: Forest plot of the multivariate evaluation of factors effecting survival, including the whole study cohort (n = 453; blood test parameters not included). Total XRT dose and fraction size are presented in Grays. P-values of statistically significant hazard ratios is in bold. XRT timing – related to timing relative to IO initiation. ECOG-PS: performance status. XRT: radiotherapy. IO: immunotherapy. Chemo: chemotherapy. Tx: treatment.

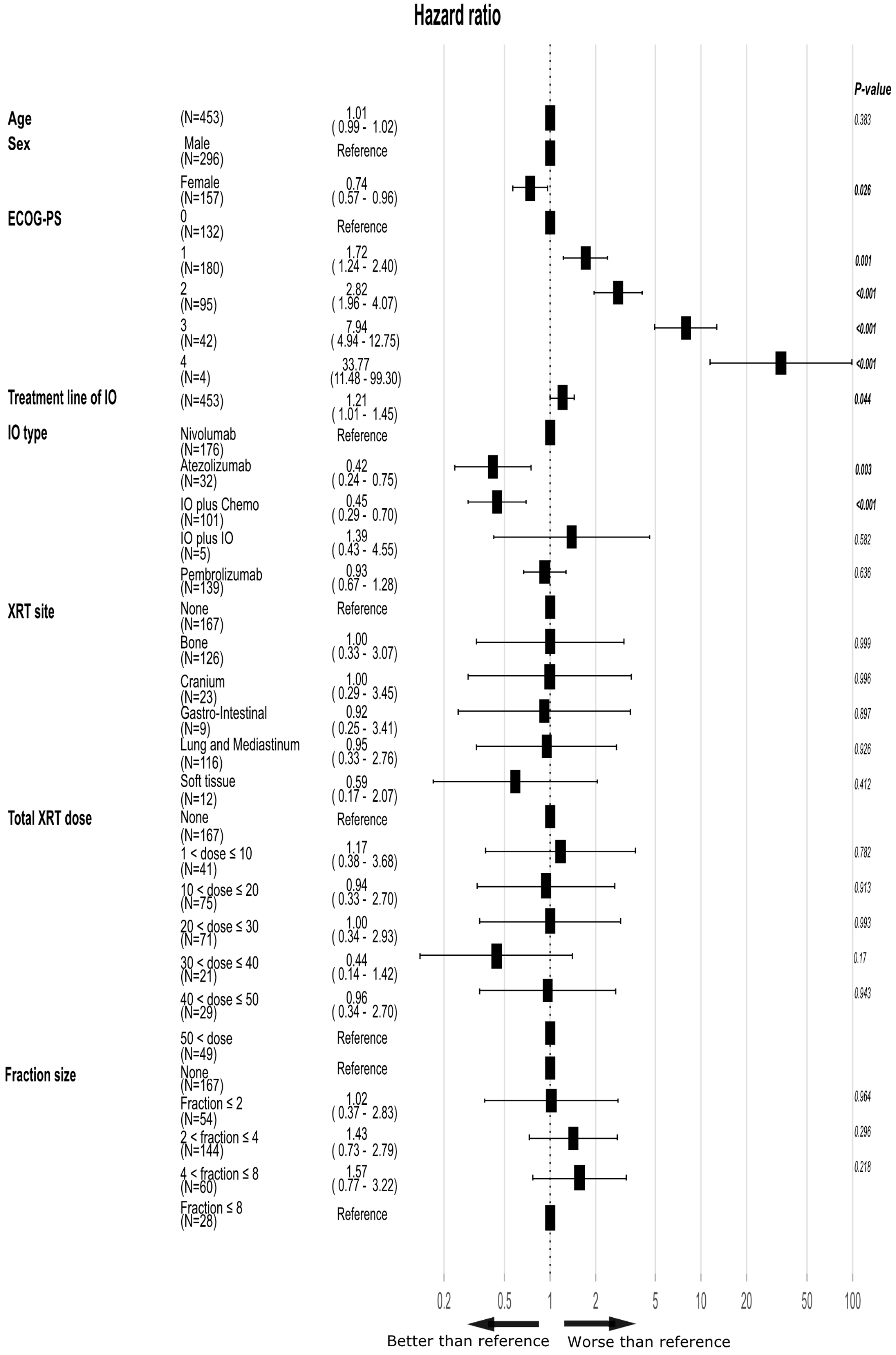

Supplement: Supplementary file 1 [file cancers-13-02800-s001.zip › Supplementary Figure S4.pdf]
