# Supplementary material for: Real-World Analysis of the Impact of Radiotherapy on Immunotherapy Efficacy in Non-Small Cell Lung Cancer
Source: Cancers (Basel). 2021 Jun 4;13(11):2800. doi: 10.3390/cancers13112800 (PMC8200093; doi:10.3390/cancers13112800)
Supplement: Supplementary file 1 [file cancers-13-02800-s001.zip › Supplementary Table S1.pdf]

Supplementary Table S1: The number of patients with the specific combination of fraction number with either total dose or faction size is indicated in each cell. The percentage in brackets relate to the total of patients that have received radiotherapy in each fraction-number subgroup.

	Number of fractions = 1<br>N (%)	Number of fractions = 2-5<br>N (%)	Number of fractions = 6-10<br>N (%)	Number of fractions >11<br>N (%)
Total XRT dose (Gy)				
1 < dose ≤ 10	41 (95.3)	-	-	-
10 < dose ≤ 20	2 (4.7)	72 (69.2)	1 (1.5)	-
20 < dose ≤ 30	-	10 (9.6)	59 (90.8)	2 (2.7)
30 < dose ≤ 40	-	6 (5.8)	2 (3.1)	13 (17.6)
40 < dose ≤ 50	-	13 (12.5)	3 (4.6)	13 (17.6)
50 < dose	-	3 (2.9)	-	46 (62.2)
Fraction size (Gy)				
Fraction ≤ 2	-	-	1 (1.5)	53 (71.6)
2 < fraction ≤ 4	2 (4.7)	60 (57.7)	61 (93.8)	21 (28.4)
4 < fraction ≤ 8	34 (79.1)	23 (22.1)	3 (4.6)	-
8 ≤ Fraction	7 (16.3)	21 (20.2)	-	-
